# Clinical decision support system supported interventions in hospitalized older patients: a matter of natural course and adequate timing

**DOI:** 10.1186/s12877-024-04823-7

**Published:** 2024-03-14

**Authors:** NA Zwietering, AEMJH Linkens, D Kurstjens, PHM van der Kuy, N van Nie-Visser, BPA van de Loo, KPGM Hurkens, B Spaetgens

**Affiliations:** 1 Department of Geriatric Medicine, Laurentius Hospital, 6040 AX Roermond, PO box 920, The Netherlands; 2 Department of Hospital Pharmacy, Erasmus Medical Centre, Rotterdam, The Netherlands; 3 Department of Internal Medicine, Division of General Internal Medicine, Section Geriatric Medicine, Maastricht University Medical Center and Cardiovascular Research Institute Maastricht, Maastricht, the Netherlands; 4 Department of Internal Medicine, Geriatric Medicine, Zuyderland Medical Centre, Heerlen/Sittard-Geleen, The Netherlands; 5 Senior Project Manager, Innovation and Funding (Scientific Research), Zuyderland Medical Centre, Heerlen, The Netherlands; 6 Digitalis Rx BV, Amsterdam, The Netherlands

**Keywords:** Medication review, Drug-related problems, Clinical Decision Support System (CDSS), Older patients, Potentially inappropriate prescribing (PIP)

## Abstract

**Background:**

Drug-related problems (DRPs) and potentially inappropriate prescribing (PIP) are associated with adverse patient and health care outcomes. In the setting of hospitalized older patients, Clinical Decision Support Systems (CDSSs) could reduce PIP and therefore improve clinical outcomes. However, prior research showed a low proportion of adherence to CDSS recommendations by clinicians with possible explanatory factors such as little clinical relevance and alert fatigue.

**Objective:**

To investigate the use of a CDSS in a real-life setting of hospitalized older patients. We aim to (I) report the natural course and interventions based on the top 20 rule alerts (the 20 most frequently generated alerts per clinical rule) of generated red CDSS alerts (those requiring action) over time from day 1 to 7 of hospitalization; and (II) to explore whether an optimal timing can be defined (in terms of day per rule).

**Methods:**

All hospitalized patients aged ≥ 60 years, admitted to Zuyderland Medical Centre (the Netherlands) were included. The evaluation of the CDSS was investigated using a database used for standard care. Our CDSS was run daily and was evaluated on day 1 to 7 of hospitalization. We collected demographic and clinical data, and moreover the total number of CDSS alerts; the total number of top 20 rule alerts; those that resulted in an action by the pharmacist and the course of outcome of the alerts on days 1 to 7 of hospitalization.

**Results:**

In total 3574 unique hospitalized patients, mean age 76.7 (SD 8.3) years and 53% female, were included. From these patients, in total 8073 alerts were generated; with the top 20 of rule alerts we covered roughly 90% of the total. For most rules in the top 20 the highest percentage of resolved alerts lies somewhere between day 4 and 5 of hospitalization, after which there is equalization or a decrease. Although for some rules, there is a gradual increase in resolved alerts until day 7. The level of resolved rule alerts varied between the different clinical rules; varying from > 50–70% (potassium levels, anticoagulation, renal function) to less than 25%.

**Conclusion:**

This study reports the course of the 20 most frequently generated alerts of a CDSS in a setting of hospitalized older patients. We have shown that for most rules, irrespective of an intervention by the pharmacist, the highest percentage of resolved rules is between day 4 and 5 of hospitalization. The difference in level of resolved alerts between the different rules, could point to more or less clinical relevance and advocates further research to explore ways of optimizing CDSSs by adjustment in timing and number of alerts to prevent alert fatigue.

**Supplementary Information:**

The online version contains supplementary material available at 10.1186/s12877-024-04823-7.

## Key points

• Describing the natural course of alerts of a Clinical Decision Support System (CDSS) provides insight in the (clinical) relevance and adequate timing of clinical rules.

• Assessing the number of alerts by and timing of clinical rules can contribute to a better understanding of how to optimize the use of a CDSS.

• By means of not running the CDSS daily on one hand and maximizing it to day 4 or 5, the total number of alerts per hospitalized patient could be significantly reduced and might therefore counteract alert fatigue.

**Supplementary Information:**

The online version contains supplementary material available at 10.1186/s12877-024-04823-7.

## Introduction

With ageing of the population, the impact of drug-related problems (DRPs) is increasing [1–5]. To reduce DRPs, especially in the setting of hospitalized older patients, CDSSs (Clinical Decision Support Systems) have been proposed as one of the major innovations that, by improving physician performance, show promising results on reducing potentially inappropriate prescribing (PIP) in hospitalized older patients [6–8]. As polypharmacy and PIP often results in negative consequences such as increased healthcare costs, and drug-related hospital admissions, it is of great importance to further investigate tools to optimise medication prescription in older people [9].

Recently, we investigated the use of a CDSS and described the real-life pattern and natural course of alerts provided by an in-hospital implemented CDSS. As such, we have shown that irrespective of an intervention by a pharmacist clinically relevant rules may become resolved and this finding may serve as an important explanation for why large clinical trials evaluating CDSS turned out negative [10]. However, in this previous study we categorized the clinical rules in 6 major groups and although this allowed us to globally study the ‘natural course’, we were unable to optimize the CDSS per specific rule.

Prior clinical trials on medication optimisation supported by a CDSS, showed a low proportion of adherence to recommendations by clinicians [11–13]. Potential determinants for this low adherence rates to the recommendations were the clinician’s opinion of little clinical relevance as well as variable attitudes towards the intervention and/or participation in clinical trials and patient-specific factors [14, 15]. It is well known that an excess of recommendations and recommendations that are less clinically relevant can contribute to alert fatigue [13, 16, 17]. However, precise figures regarding the frequency of alert reporting and whether there exists an ideal frequency and timing remain elusive. Moreover, in previous study protocols the CDSS supported intervention was performed at a single timepoint within 48-72 h of hospitalization [16, 18, 19]. Therefore, in this study we aim: (I) to describe the natural course and interventions based on the top 20 of generated alerts over time in a hospitalized population (from day 1 to 7); and (II) to explore whether an ideal timing and frequency can be defined (in terms of day per rule).

## Methods

### Setting and patients

This retrospective study was performed in Zuyderland Medical Centre, a large teaching hospital in the Netherlands. In the year 2018, all hospitalized patients, aged ≥ 60 years were included with the exception of patients admitted to rehabilitation and short stay departments. We collected demographic data (age and sex). The evaluation of the CDSS was investigated in a retrospective study using an anonymized database used for standard care, which is why this study did not require ethical approval.

### Clinical decision support system (CDSS)– clinical rule reporter (CRR)

The CDSS used in this study is the Clinical Rule Reporter (CRR), which has been developed in Zuyderland Medical Centre and has been implemented in daily practice since 2016. The CDSS runs daily and is used for medication surveillance of all hospitalized patients. It combines demographic information (e.g. age), laboratory values (e.g. kidney function (eGFR)) and medication-related information (e.g. dosage) to obtain specific alerts based on clinical rules (i.e. algorithms) [20, 21]. The clinical rules cover dosage adjustments in decreased renal function or based on higher age, electrolyte dysfunction in relation to medication, anticoagulation therapy and INR, long use of antibiotic therapy, intravenous to oral switch in antibiotic therapy, and use of opioids without laxative agents. Whenever a laboratory value is below or above a set limit, in combination with the appropriate drug, the CDSS will generate an alert. The cut-off value for the laboratory values to create an alert are: eGFR-MDRD < 50 ml/min, potassium > 5.5 or < 3.0 mmol/l and INR > 5. In this study, the CDSS had 80 different rules in use (supplementary data Table S1).

### Data collection and analysis

The clinical pharmacist receives daily reports detailing patient-specific alerts that require action, known as ‘red alerts’. These ‘red alerts’ signal significant concerns or issues that require a follow-up action by the CDSS (‘unresolved’) and potentially further intervention. Additionally, there are ‘green alerts’, that do not require follow-up action (‘resolved’). For this study, ‘an intervention by the clinical pharmacist’ was defined as having a rule discussed in detail (i.e. consultation between clinical pharmacist and attending physician) or when an intervention was initiated by the clinical pharmacist or attending physician (after such consultation).

All data on generated alerts and subsequent interventions were extracted into Qlik Sense version September 2020 SR1. This is a tool which visualizes data in an interactive way. We collected data on (1) the total number of ‘red alerts’, (2) the total number of ‘red alerts’ per rule of the top 20 rules (representing the 20 rules that most frequently generated red alerts), (3) the number of top 20 rule alerts resulting in an action or no action of the pharmacist and (4) the outcomes of the alerts of the top 20 rules, described as ‘green’ (resolved), ‘red’ (unresolved) and ‘unknown’. All data were collected on 7 consecutive days (day 1 to 7 of hospitalization).

**Fig. 1:**
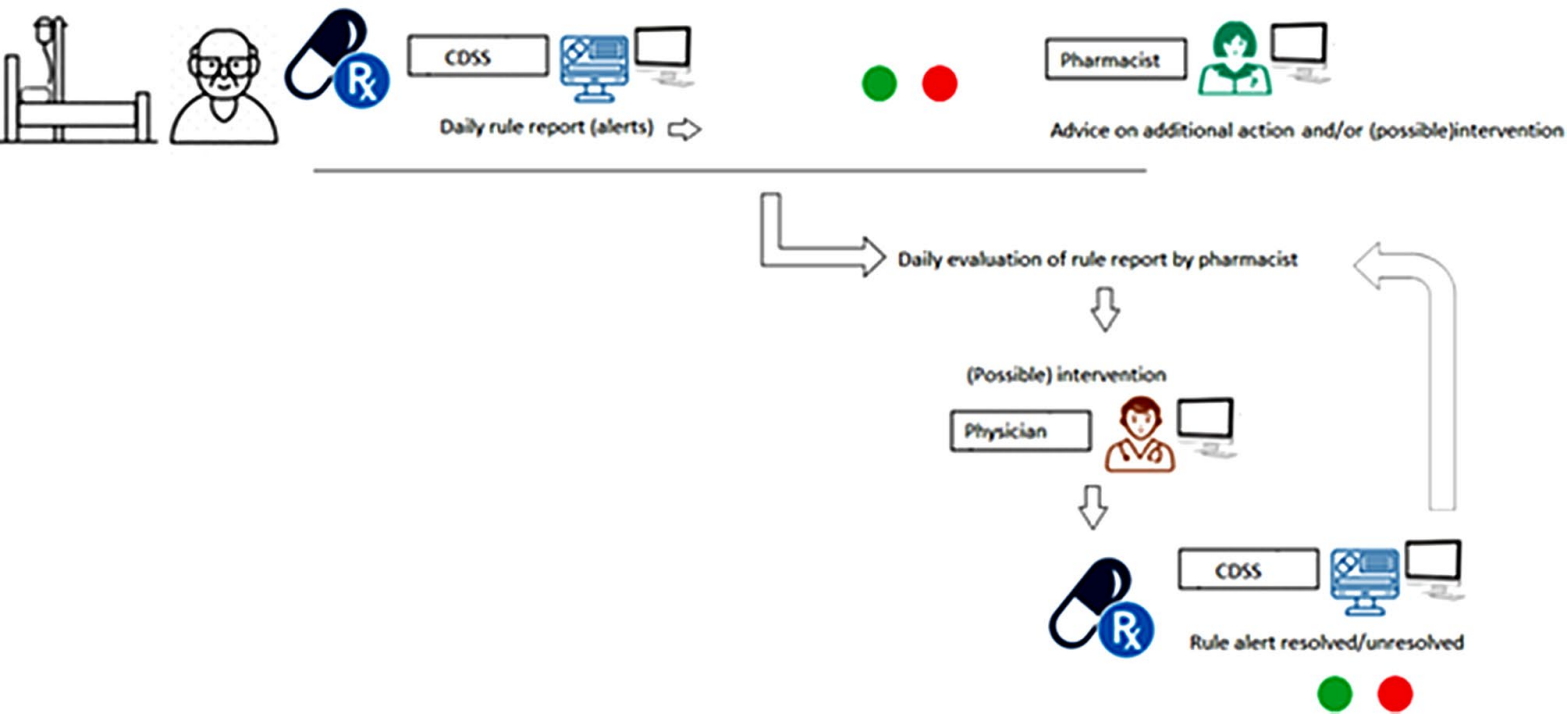
Schematic overview of the CDSS. *Legend* The CDSS generates a report for each patient daily, in which all the rules of the CDSS are assessed. All rules receive either a red or green outcome, with red alerts requiring action and attention, and they are evaluated by the pharmacist. The pharmacist then assesses whether action is needed for the healthcare provider and communicates this. The CDSS runs daily, and all rules/alerts are once again categorized as red or green. When a red alert remains red, we define it as an “unresolved” alert, and if the rule turns green, we define it as a “resolved” alert. We evaluated the rules and outcomes for the first 7 days of admission

We analyzed the change in color of the alerts to evaluate whether a patient-specific alert has been resolved or not. On the first day of de study (day 0), all evaluated alerts were ‘red’. From day 1 onward, the status of these alerts could have changed to ‘green’ (resolved) or ‘red’ (unresolved) and during the 6 consecutive days will change depending on whether there is new information that will affect the clinical rule, such as new laboratory results (i.e. normalization of laboratory values) or a change in medication dosage. As a result, the pharmacist repeatedly evaluates a certain rule during the 7-day hospitalization, based on the current alert status. Actions or interventions follow as necessary, sometimes after consultation with a physician. Figure 1 represents a schematic overview of the CDSS. We analyzed the percentage of resolved (‘green’) and unresolved (‘red’) alerts, excluding ‘unknown’ alerts. These ‘unknown’ alerts were omitted because it was unclear if they were resolved due to medication discontinuation (making the rule irrelevant) or due to patient discharge. If a specific rule only had ‘unknown’ results, the percentage of resolved rules decreased to 0%, and the representation of that rule in our figures concluded prematurely. This approach allows us to illustrate the natural progression of the generated alerts for the top 20 clinical rules (without pharmacist intervention) and the progression after pharmacist intervention from days 1 to 7 of hospitalization.

Descriptive statistics were used to describe the study population and were presented as means (± standard deviation (SD)) or percentages (%), and Chi-square of Fisher’s exact test were used as appropriate. Statistics were performed using SPSS statistics v.28 (IBM, Armonk, New York, USA).Charts were created with Excel.

## Results

In 2018, anonymised data from 3574 unique hospitalized patients were included for this study. The mean age was 76.7 (SD 8.3) years and 53% were female. From these patients, in total 8073 red alerts were generated, of which 7907 (97.9% of total) were handled by the pharmacist (day one). With the top 20 of rule alerts we covered roughly 90% of the total number of red alerts. The demographic characteristics and subdivision of total number of top 20 rule alerts on day 1 and day 7 are described in Table 1.

**Table 1:** Baseline characteristics

Unique patients (*n* = 3754)			
Age (years), mean (SD)	76.7 (8.3)		
Male, n (%)	1764 (47.0)		
*Rule alerts, n (%)*	Day one		Day seven
*Total*	7907 (100)		5261 (100)
*Total number of top 20 rule alerts, n (%)*	7156 (90.5)		4757 (90.4)
Top 20 of rule alerts, n (%)*			
Potassium levels	2170 (30.3)		1418 (30.0)
MDRD required	942 (13.2)		885 (18.6)
Anticoagulation therapy and INR	587 (8.2)		384 (8.1)
Renal dysfunction + Levetiracetam	428 (6.0)		185 (3.9)
Long use antibiotic therapy	366 (5.1)		322 (6.8)
Renal dysfunction + Tazocin/Piperacillin	346 (4.8)		149 (3.1)
Renal dysfunction + Cefazolin	266 (3.7)		211 (4.4)
Potassium levels + Digoxin	247 (3.5)		189 (4.0)
Opioids without laxative agents	225 (3.1)		201 (4.2)
Renal dysfunction + Barnidipine	195 (2.7)		95 (2.0)
Renal dysfunction + Valaciclovir	175 (2.4)		71 (1.5)
Renal dysfunction + Benzylpenicillin	166 (2.3)		56 (1.2)
Renal dysfunction + Amoxicillin	147 (2.0)		53 (1.1)
Intravenous to oral switch- Metronidazole	145 (2.0)		136 (2.9)
Renal dysfunction + Sucralfate	141 (2.0)		47 (1.0)
Renal dysfunction + Pramipexole	139 (1.9)		64 (1.3)
Intravenous to oral switch -Flucloxacillin	129 (1.8)		97 (2.0)
Renal dysfunction + Meropenem	97 (1.4)		38 (0.8)
Renal dysfunction + amoxicillin/clavulanic acid	96 (1.3)		75 (1.6)
Renal dysfunction + Tranexamic acid	84 (1.2)		71 (1.5)

* percentage is the division of the individual alerts per rule divided by total number of alerts in top 20

**Fig. 2:**
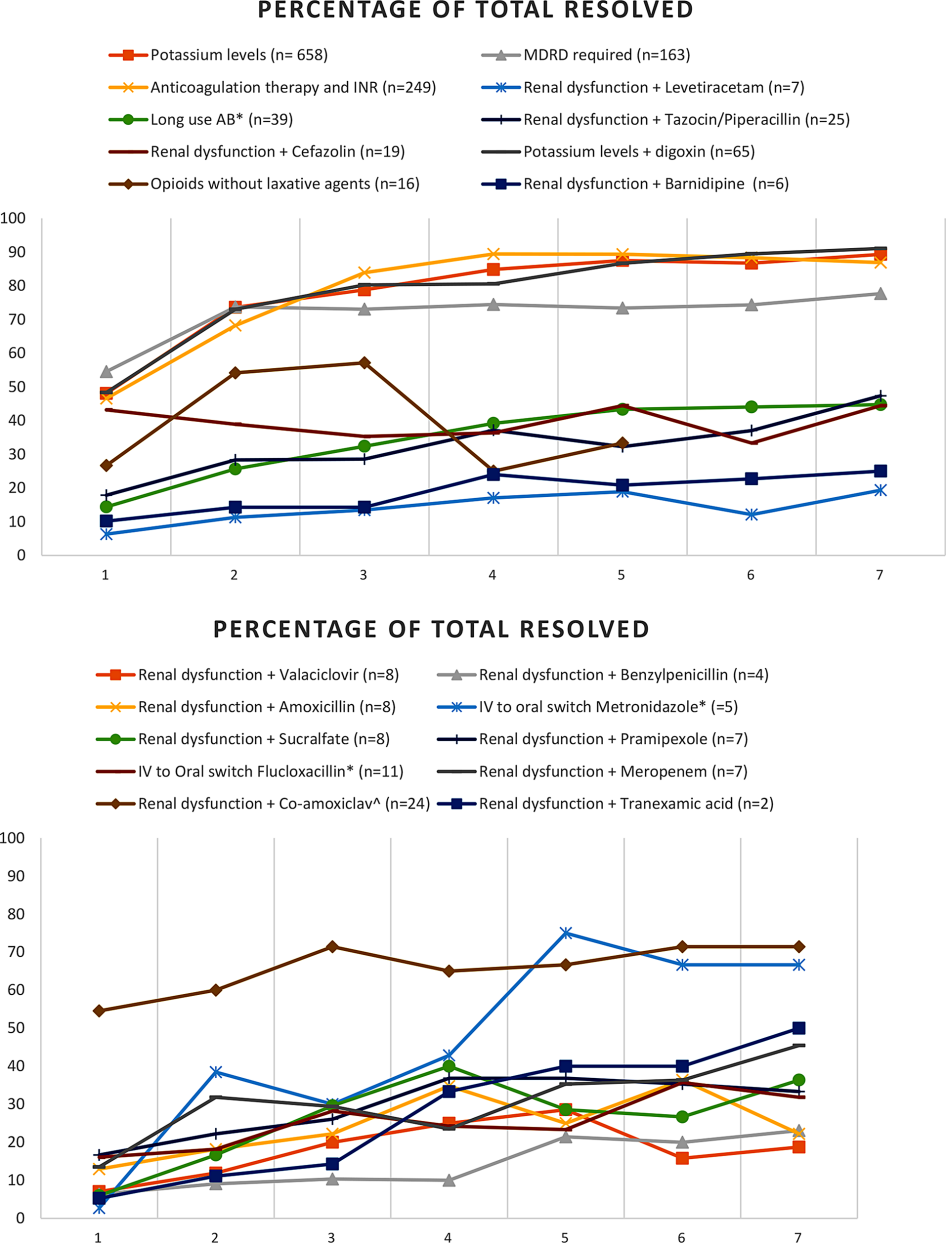
Alerts with intervention: rules 1 to 10 (percentage of total resolved ). *AB = antibiotics. ^co-amoxiclav = amoxicillin/clavulanic acid - *IV = intravenous

In our figures, we presented the proportion of resolved alerts, and we specified the highest number of alerts for each clinical rule. Detailed data on the exact count of resolved alerts is available in the Supplementary Data (Table S2). The percentages of resolved alerts (top 10) of those with an intervention of the clinical pharmacist are shown in Fig. 2a. As such, one can see that on average for most rules the highest percentage of resolved alerts lies somewhere between day 4 and 5 of hospitalization. Nevertheless, there are rules for which the percentages keep gradually increasing until day 7, such as potassium + digoxin and long use antibiotics. For other rules, such as opioids without laxatives the percentages of resolved alerts are drastically decreased after day 3 or more or less equal from day 1 to 7, such as for renal dysfunction + barnidipine and renal dysfunction + levetiracetam. There is also a clear difference in the level of resolved alert percentages between the different rules; varying from > 70% for potassium levels (+ digoxin), anticoagulation therapy and INR, and MDRD requirement to < 25% for renal dysfunction + barnidipine and renal dysfunction + levetiracetam.

The percentages of resolved alerts (top 11–20) of those with an intervention of the clinical pharmacist are shown in Fig. 2b. Again, for most rules the highest percentage of resolved alerts lies somewhere between day 4 and 5 of hospitalization, after which there is equalization. Although for some rules, such as renal dysfunction + meropenem and renal dysfunction + tranexamic acid, there is a gradual increase until day 7. For renal dysfunction + amoxicillin-clavulanic acid and intravenous to oral switch of metronidazole the percentage of resolved alerts is > 60%, whereas this does not exceed 25% in renal dysfunction + valaciclovir and renal dysfunction + benzylpenicillin.

**Fig. 3:**
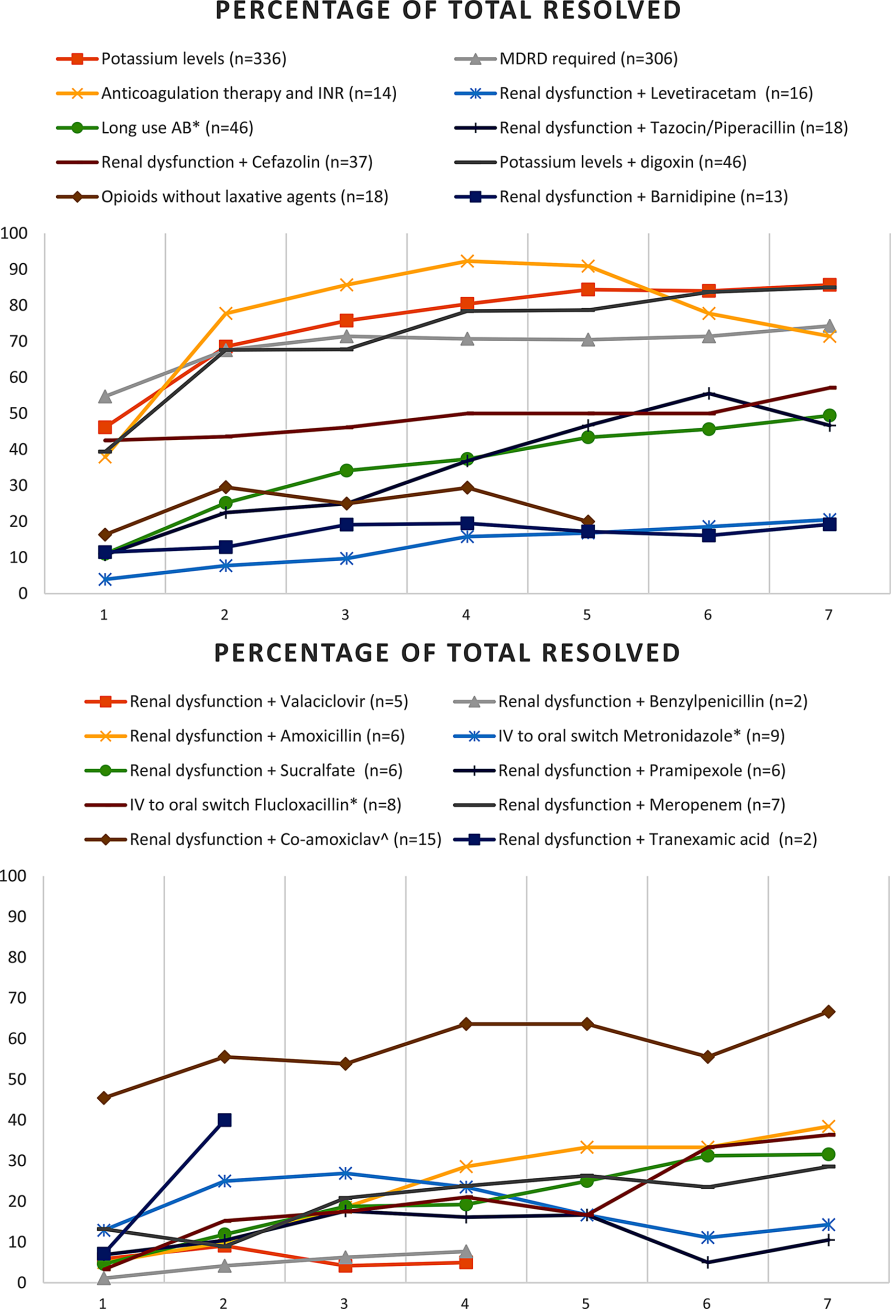
Alerts without intervention: rules 1 to 10 (percentage of total resolved). *AB = antibiotics. ^co-amoxiclav = amoxicillin/clavulanic acid - *IV = intravenous

The percentages of resolved alerts (top 10) of those without an intervention of the clinical pharmacist are shown in Fig. 3a. As is the case for the resolved alerts with an intervention, for most rules the highest percentage of resolved alerts lies somewhere between day 4 and 5 of hospitalization, after which there is equalization or a decrease. Although for some rules, such as long use antibiotics and renal dysfunction + cefazolin, there is a gradual increase until day 7. The lines that represent alerts that dropped to 0% (meaning that all alerts are unknown due to medication discontinuation or the patient has been discharged) end early in the figure. There is also a clear difference in the level of resolved percentages between the different rules; varying from > 70% for potassium levels (+ digoxin), anticoagulation therapy and INR, and MDRD requirement to < 20% for renal dysfunction + barnidipine and renal dysfunction + levetiracetam.

The percentages of resolved alerts (top 11–20) of those without an intervention of the clinical pharmacist are shown in Fig. 3b. As goes for the other figures, for most rules the highest percentage of resolved alerts lies somewhere between day 4 and 5 of hospitalization with the exception of renal dysfunction + amoxicillin/clavulanic acid, renal dysfunction + sucralfate, and renal dysfunction + amoxicillin; where there is a gradual increase until day 7. The lines that represent alerts that dropped to 0% (meaning that all alerts are unknown due to medication discontinuation or the patient has been discharged) end early in the figure. The level of resolved alerts was > 50% for renal dysfunction + amoxicillin/clavulanic acid and < 50% for all other rule alerts.

For most clinical rules there were no differences between the number of resolved rule alerts whether the pharmacist intervened or not. We did however notice a difference concerning potassium levels on day 2 (73.6% with vs. 68.6% without pharmacist intervention, *p* = 0.05); IV switch to oral flucloxacillin on day 1 (16.1% with vs. 3.3% without pharmacist intervention, *p* = 0.03); renal dysfunction + meropenem on day 2 (31.8% with vs. 8.9% without pharmacist intervention, *p* = 0.03); renal dysfunction + levetiracetam on day 3 (13.5% with vs. 9.8% without pharmacist intervention, *p* = 0.03) and renal dysfunction + pramipexole on day 6 (35.3% with vs. 5% without pharmacist intervention, *p* = 0.03).

## Disscusion

This study demonstrates the course of the 20 most frequently generated alerts of an in-hospital implemented CDSS that was run daily during hospitalization in a large cohort of older patients. As such, for most rules in top 20, we have found that the percentages of resolved alerts gradually increases per day and for most rules the highest percentage of resolved alerts lied between day 4 and 5 of hospitalization. Some rules however had a gradual increase until day 7 of hospitalization. There is a great difference between the level of resolved alerts between the different rules, that suggests that certain rules are regarded as more or less clinically relevant in the field of medication and patient safety. For the top 20 rules, the most resolved alerts were among those concerning potassium levels, anticoagulation therapy and requirement for MDRD; that seems logical given the degree of potential clinical impact (e.g. bleeding risk or cardiac arrhythmias). A significant number of the top 20 rules concern renal dysfunction and the use of various medications, especially antibiotics. This is worth nothing, as clinicians often will tolerate moderate renal function decline for a short duration to facilitate the action of other medications and clinical relevance could therefore impact the number of resolved rules. However, we observed little difference in the number of resolved rules and the ideal number of days with the highest percentage of resolved rules, whether a pharmacist evaluated the rule or not [10].

Although the gradually increasing percentages per day are not surprising at all, we believe this finding is of interest. One might argue that only small increases after day 4 or 5 in daily practice may not be (clinically) relevant. We therefore propose that for rules considered clinically relevant (by the clinical pharmacologist and physician) these rule reports should be run daily, but for others, an ideal timing on day 4 or 5 could be suggested. Previous research showed that approximately 74% of recommendations based on STOPP (Screening Tool of Older Persons’ Prescriptions) and START (Screening Tool to Alert to Right Treatment) criteria by a CDSS were considered to be clinically relevant; varying for ‘possible low’ to ‘possibly very important’ relevance [14, 22]. The remaining quarter of recommendations was considered of ‘no clinical relevance’ (21.5%) or possible ‘adverse significance’ (5%) [14]. Theoretically, excluding the clinical rules considered to be of no (or adverse) clinical relevance could lead to a reduction of 25% of total alerts, and improve adherence to recommendations. Hence, providing only clinical relevant recommendations or prioritizing them, could help reduce the phenomenon of alert fatigue [23]. By means of not running the CDSS daily on one hand and maximizing it to day 4 or 5, or trigger it only twice, on day 1 and on day 4 or 5, the total number of alerts per hospitalized patient could be significantly reduced and might therefore also counteract alert fatigue. Although this is highly speculative, it is very likely the number and frequency and relevance of alerts is an important contributor to alert fatigue and improving the functionality of a CDSS in an ageing population could be of great significance. Additionally, in the results, we observe that for several rules, the number of resolved rules decreases at a certain point. This is partly influenced by the fact that the number of unknown rules increases over time. The reason for an unknown rule is the discontinuation of medication or patient discharge. While we are unable to categorize this “unknown” group as resolved or unresolved, it does impact our results. As such, it may potentially explain the declining trend after a few days for all rules. And for some rules, such as laxatives and opioids, this might even explain why the percentage drops to zero. These patients may not all have been discharged, but it is possible that opioids were discontinued, making the problem resolved.

We also observed that there was little difference in the optimal outcome between whether or not the pharmacist intervened. Since we do not have data on the specific advice given by the pharmacist, it is difficult to assess why recommendations were not followed. Even though the main focus of this study was not to evaluate the pharmacist’s impact on resolving alerts, it is noteworthy that significant differences, if present, typically occurred within the first three days. This could account for the lack of notable differences observed subsequently, and also suggests the pharmacist’s early identification of the clinical issues in question. Most of these clinical rules (with the exception of potassium levels) however, concerned a small number of rule alerts, making it difficult to interpret the relevance of these differences. These aspects should be explored in future research. For example, it could be due to a certain time window that is necessary to resolve a medication issue, as is the case for potassium supplementation. Alternatively, it could be related to healthcare providers independently recognizing the same problems as those incorporated in the rules of this CDSS, resulting in no improvement or shift in the ideal frequency or timing.

Our study has strengths and limitations that need to be discussed. Strengths include the large sample size, the real-life clinical setting of an already implemented CDSS and the fact this is studied for the first time. Nevertheless, our study also has some limitations. First, our study is limited by its retrospective and observational design. Second, in 2018 our CDSS only consisted of 80 clinical rules. As such, clinically relevant, but also well-known rules (such as START/STOPP) have not been included in this version of the CDSS, making this study more difficult to interpret in the current field of studies investigating generic CDSSs. Nevertheless, we believe by investigating the top 20 (most frequently generated alerts) and alert fatigue as important generic aspect and quality of CDSS, we provide insight in how we could optimize the functionality of a CDSS. It is important to emphasize that we refrain from speculating that these optimizations will necessarily result in a direct reduction in the number of DRPs. Third, as previously mentioned, the group of unknown alerts impacts the results; however, it is challenging to predict the specific manner in which the results are affected. Fourth, although this is a relatively large study, we only had access to very few clinical data and therefore we were unable to investigate the impact of clinical predictors. Despite this, we have included a cohort of patients in which DRPs are of particular interest, namely a population with a mean age of 76 years, which is often excluded in clinical trials.

## Conclusions

This study demonstrated for the first time that for most clinical rules by a CDSS, irrespective of an intervention by the pharmacist, the highest percentages of resolved alerts were reached between day 4 and 5 of hospitalization. As such, we have shown that it might be profitable not running the CDSS daily, to significantly reduce the total number of alerts per hospitalized patient. Moreover it seems profitable to discuss clinical relevance of CDSS recommendations, given the difference in adherence to recommendations, also to reduce the number of alerts. Lowering the number and frequency of alerts could diminish alert fatigue and improve the functionality of a CDSS. Future research should focus on optimizing quantity, clinical relevance and timing of recommendations by a CDSS on patient related outcomes.

### Electronic supplementary material

Below is the link to the electronic supplementary material.

Supplementary Material 1

## Data Availability

Data are available on request from the lead author (NA Zwietering).
