# Supplementary material for: Clinical decision support system supported interventions in hospitalized older patients: a matter of natural course and adequate timing
Source: BMC Geriatr. 2024 Mar 14;24:256. doi: 10.1186/s12877-024-04823-7 (PMC10941377; doi:10.1186/s12877-024-04823-7)
Supplement: Supplementary file 1 — Supplementary Material 1 [file 12877_2024_4823_MOESM1_ESM.docx]

**Supplementary Material**

**Title**

Clinical Decision Support System supported interventions in hospitalized older patients: a matter of natural course and adequate timing

**Author list**

NA Zwietering^1,2^, AEMJH Linkens^3,2^ , D Kurstjens^4^, PHM van der Kuy^2^, N van Nie-Visser ^5^, BPA van de Loo^6^, KPGM Hurkens^4^, B Spaetgens^3^

^1^ Department of Geriatric Medicine, Laurentius Hospital, Roermond, The Netherlands

^2^ Department of Hospital Pharmacy, Erasmus Medical Centre, Rotterdam, The Netherlands.

^3^ Department of Internal Medicine, Division of General Internal Medicine, Section Geriatric Medicine, Maastricht University Medical Center and Cardiovascular Research Institute Maastricht, Maastricht, the Netherlands.^4^ Department of Internal Medicine, Geriatric Medicine, Zuyderland Medical Centre, Heerlen/Sittard-Geleen The Netherlands.

^5^ Senior Project Manager, Innovation and Funding (Scientific Research), Zuyderland Medical Centre, Heerlen, The Netherlands.

^6^ Digitalis Rx BV, Amsterdam, The Netherlands

**Table S1. Overview of the clinical rules top 20**

|  | **Title of the rule** |
| --- | --- |
| 1 | Potassium levels |
| 2 | MDRD required |
| 3 | Anticoagulation therapy and INR |
| 4 | Renal dysfunction + Levetiracetam |
| 5 | Long use antibiotic therapy |
| 6 | Renal dysfunction + Tazocin/Piperacillin |
| 7 | Renal dysfunction + Cefazolin |
| 8 | Potassium levels + Digoxin |
| 9 | Opioids without laxative agents |
| 10 | Renal dysfunction + Barnidipine |
| 11 | Renal dysfunction + Valaciclovir |
| 12 | Renal dysfunction + Benzylpenicillin |
| 13 | Renal dysfunction + Amoxicillin |
| 14 | IV to oral switch Metronidazole |
| 15 | Renal dysfunction + Sucralfate |
| 16 | Renal dysfunction + Pramipexole |
| 17 | IV to oral switch Flucloxacillin |
| 18 | Renal dysfunction + Meropenem |
| 19 | Renal dysfunction + amoxicillin/clavulanic acid |
| 20 | Renal dysfunction + Tranexamic acid |

**Table S2. Number of resolved alerts with pharmacist (Ph+) or without (Ph-) intervention, n (% of total without unknown)**

|  | **Day 1** | | | **Day 2** | | | **Day 3** | | | **Day 4** | | | **Day 5** | | | **Day 6** | | | **Day 7** | | |
| --- | --- | --- | --- | --- | --- | --- | --- | --- | --- | --- | --- | --- | --- | --- | --- | --- | --- | --- | --- | --- | --- |
| **Rule ID** | Ph+ | Ph- | p | Ph+ | Ph- | P | Ph+ | Ph- | p | Ph+ | Ph- | p | Ph+ | Ph- | p | Ph+ | Ph- | p | Ph+ | Ph- | p |
| **Potassium levels** | 619 (48.0) | 336 (46.2) | 0.44 | 658 (73.6) | 336 (68.6) | *0.05* | 613 (78.8) | 319 (75.8) | 0.24 | 576 (84.8) | 304 (80.4) | 0.86 | 546 (87.5) | 287 (84.4) | 0.20 | 496 (86.7) | 263 (84.0) | 0.31 | 468 (89.3) | 246 (85.7) | 0.14 |
| **MDRD required** | 163 (54.5) | 306 (54.7) | 0.89 | 160 (73.7) | 271 (67.6) | 0.12 | 122 (73.0) | 210 (71.4) | 0.75 | 102 (74.5) | 162 (70.7) | 0.47 | 91 (73.3) | 141 (70.5) | 0.61 | 84 (74.3) | 125 (71.4) | 0.69 | 87 (77.7) | 110 (74.3) | 0.56 |
| **Anticoagulation therapy and INR** | 231 (46.6) | 11 (37.9) | 0.44 | 249 (68.2) | 14 (77.8) | 0.45 | 224 (83.9) | 12 (85.7) | 1.00 | 220 (89.4) | 12 (92.3) | 1.00 | 193 (89.3) | 10 (90.9) | 1.00 | 174 (88.3) | 7 (77.8) | 0.30 | 159 (86.9) | 5 (71.4) | 0.25 |
| **Renal dysfunction + Levetiracetam** | 6 (6.3) | 12 (4.0) | 0.40 | 7 (11.3) | 14 (7.8) | 0.44 | 7 (13.5) | 13 (9.8) | *0.03* | 7 (17.0) | 16 (15.8) | 0.42 | 7 (18.9) | 16 (16.8) | 0.80 | 4 (12.1) | 16 (18.6) | 0.58 | 6 (19.4) | 12 (20.5) | 1.00 |
| **Long use AB** | 20 (14.4) | 20 (11.0) | 0.40 | 29 (25.7) | 34 (25.2) | 1.00 | 34 (32.4) | 42 (34.2) | 0.89 | 38 (39.2) | 40 (37.4) | 0.89 | 39 (43.3) | 46 (43.4) | 1.00 | 37 (44.0) | 42 (45.7) | 0.88 | 34 (44.7) | 44 (49.4) | 0.63 |
| **Renal dysfunction + Tazocin/Piperacillin** | 25 (17.9) | 17 (10.8) | 0.10 | 21 (28.4) | 18 (22.5) | 0.46 | 16 (28.6) | 12 (25.0) | 0.83 | 13 (37.1) | 14 (36.8) | 1.00 | 10 (32.3) | 14 (46.7) | 0.30 | 10 (37.0) | 10 (55.6) | 0.24 | 9 (47.4) | 7 (46.7) | 1.00 |
| **Renal dysfunction + Cefazolin** | 19 (43.2) | 37 (42.5) | 1.00 | 7 (38.9) | 17 (43.6) | 0.78 | 6 (35.3) | 12 (46.2) | 0.54 | 4 (36.4) | 12 (50.0) | 0.49 | 4 (44.4) | 9 (50.0) | 1.00 | 3 (33.3) | 8 (50.0) | 0.68 | 4 (44.4) | 8 (57.1) | 0.68 |
| **Potassium levels + digoxin** | 58 (48.3) | 39 (39.4) | 0.22 | 65 (73.0) | 46 (67.7) | 0.48 | 61 (80.3) | 40 (67.8) | 0.11 | 58 (80.6) | 40 (78.4) | 0.82 | 52 (86.7) | 37 (78.7) | 0.31 | 51 (89.5) | 36 (83.7) | 0.76 | 51 (91.1) | 34 (85.0) | 0.52 |
| **Opioids without laxative agents** | 16 (26.7) | 18 (16.4) | 0.11 | 13 (54.2) | 13 (29.6) | *0.07* | 8 (57.1) | 6 (25.0) | *0.08* | 1 (25.0) | 5 (29.4) | 1.00 | 1 (33.3) | 3 (20.0) | 1.00 | 0 | 0 | 1.00 | 0 | 0 | 1.00 |
| **Renal dysfunction + Barnidipine** | 6 (10.2) | 13 (11.5) | 1.00 | 5 (14.3) | 8 (12.9) | 1.00 | 4 (14.3) | 9 (19.2) | 0.76 | 6 (24.0) | 8 (19.5) | 0.76 | 5 (20.8) | 5 (17.2) | 1.00 | 5 (22.7) | 5 (16.1) | 0.72 | 5 (25.0) | 5 (19.2) | 0.73 |
| **Renal dysfunction + Valaciclovir** | 5 (7.0) | 5 (5.9) | 1.00 | 5 (11.9) | 4 (9.1) | 0.74 | 7 (20.0) | 1 (4.2) | 0.12 | 7 (25.0) | 1 (5.0) | 0.12 | 8 (28.6) | 0 | 1.00 | 3 (15.8) | 0 | 1.00 | 3 (18.8) | 0 | 1.00 |
| **Renal dysfunction + Benzylpenicillin** | 4 (6.4) | 1 (1.1) | *0.07* | 3 (9.1) | 2 (4.2) | 0.38 | 3 (10.3) | 2 (6.3) | 0.67 | 2 (10.0) | 2 (7.7) | 1.00 | 3 (21.4) | 0 | 1.00 | 3 (20.0) | 0 | 1.00 | 3 (23.1) | 0 | 1.00 |
| **Renal dysfunction + Amoxicillin** | 7 (13.0) | 4 (5.0) | 0.12 | 6 (18.2) | 4 (10.0) | 0.33 | 6 (22.2) | 5 (18.5) | 1.00 | 8 (34.8) | 6 (28.6) | 0.75 | 4 (25.0) | 5 (33.3) | 0.70 | 4 (36.4) | 5 (33.3) | 1.00 | 2 (22.2) | 5 (38.5) | 0.65 |
| **IV switch to oral Metronidazole** | 1 (2.7) | 8 (12.9) | 0.15 | 5 (38.5) | 9 (25.0) | 0.48 | 3 (30.0) | 7 (26.9) | 1.00 | 3 (42.9) | 4 (23.5) | 0.37 | 3 (75.0) | 2 (16.7) | *0.06* | 2 (66.7) | 1 (11.1) | 0.13 | 2 (66.7) | 1 (14.3) | 0.18 |
| **Renal dysfunction + Sucralfate** | 3 (6.0) | 4 (4.6) | 0.70 | 5 (16.7) | 5 (12.0) | 0.73 | 8 (29.6) | 6 (18.8) | 0.37 | 8 (40.0) | 5 (19.2) | 0.19 | 6 (28.6) | 5 (25.0) | 1.00 | 4 (26.7) | 5 (31.3) | 1.00 | 4 (36.4) | 6 (31.6) | 1.00 |
| **Renal dysfunction + Pramipexole** | 7 (16.7) | 6 (6.9) | 0.12 | 6 (22.2) | 5 (10.4) | 0.18 | 6 (26.1) | 6 (17.7) | 0.52 | 7 (36.8) | 5 (16.1) | 0.17 | 7 (36.8) | 5 (16.7) | 0.17 | 6 (35.3) | 1 (5.0) | *0.03* | 5 (33.3) | 2 (10.5) | 0.11 |
| **IV switch to oral Flucloxacillin** | 9 (16.1) | 2 (3.3) | *0.03* | 8 (18.2) | 7 (15.2) | 0.78 | 11 (28.2) | 7 (17.5) | 0.29 | 8 (24.2) | 8 (21.5) | 1.00 | 7 (23.3) | 5 (16.7) | 0.75 | 10 (35.7) | 8 (33.3) | 1.00 | 7 (31.8) | 8 (36.4) | 1.00 |
| **Renal dysfunction + Meropenem** | 5 (13.5) | 7 (13.2) | 1.00 | 7 (31.8) | 5 (8.9) | *0.03* | 5 (29.4) | 5 (20.8) | 0.71 | 4 (23.5) | 5 (23.8) | 1.00 | 6 (35.3) | 5 (26.3) | 0.72 | 4 (36.7) | 4 (23.5) | 0.67 | 5 (45.5) | 4 (28.6) | 0.38 |
| **Renal dysfunction + amoxicillin/ clavulanic acid** | 24 (54.6) | 15 (45.5) | 0.64 | 15 (60.0) | 10 (55.6) | 1.00 | 15 (71.4) | 7 (53.9) | 0.46 | 13 (65.0) | 7 (63.6) | 1.00 | 10 (66.7) | 7 (63.6) | 1.00 | 10 (71.4) | 5 (55.6) | 0.66 | 10 (71.4) | 4 (66.7) | 1.00 |
| **Renal dysfunction + Tranexamic acid** | 1 (5.3) | 1 (7.1) | 1.00 | 1 (11.1) | 1 (7.1) | 1.00 | 1 (14.3) | 0 | 1.00 | 2 (33.3) | 0 | 1.00 | 2 (40.0) | 0 | 1.00 | 2 (40.0) | 0 | 1.00 | 2 (50.0) | 0 | 1.00 |

p= p-value
